# Supplementary material for: Mutation spectrum of NOD2 reveals recessive inheritance as a main driver of Early Onset Crohn’s Disease
Source: Sci Rep. 2021 Mar 10;11:5595. doi: 10.1038/s41598-021-84938-8 (PMC7946957; doi:10.1038/s41598-021-84938-8)
Supplement: Supplementary file 1 — Supplementary Information 1. [file 41598_2021_84938_MOESM1_ESM.pdf]

Supplementary Information for:

Mutation spectrum of *NOD2* reveals recessive inheritance as a main driver of Early Onset Crohn's Disease

Julie E. Horowitz, Ph.D.[1], Neil Warner, Ph.D.[2, 3], Jeffrey Staples, Ph.D.[1], Eileen Crowley, M.D.[2,3], Nehal Gosalia, Ph.D.[1], Ryan Murchie, M.Sc.[2,3], Cristopher Van Hout, Ph.D.[1], Karoline Fiedler[2,3], Gabriel Welch[1], Alejandra Klauer King, Ph.D.[1], Jeffrey G. Reid, Ph.D.[1], John D. Overton, Ph.D.[1], Aris Baras, M.D.[1], Alan R. Shuldiner, M.D.[1], Anne Griffiths, M.D.[2], Omri Gottesman, M.D.[1], Aleixo M. Muise, M.D., Ph.D.[2, 3, 4, *], Claudia Gonzaga-Jauregui, Ph.D.[1, *]

[1] Regeneron Genetics Center, Regeneron Pharmaceuticals Inc., Tarrytown, NY, USA

[2] SickKids Inflammatory Bowel Disease Center, Hospital for Sick Children, Toronto, ON, Canada

[3] Cell Biology Program, Research Institute, Hospital for Sick Children, Toronto, ON, Canada.

[4] Departments of Pediatrics and Biochemistry, University of Toronto, Toronto, ON, Canada.

*Co-corresponding authors

Figure S1. Example pedigrees for homozygous and compound heterozygous *NOD2* carriers in an early onset IBD cohort. Example pedigrees *NOD2* variants identified in families with pediatric onset IBD. Diagnosis and age at diagnosis are indicated for the affected probands (filled dark grey symbols); unaffected relatives are depicted as unfilled symbols.

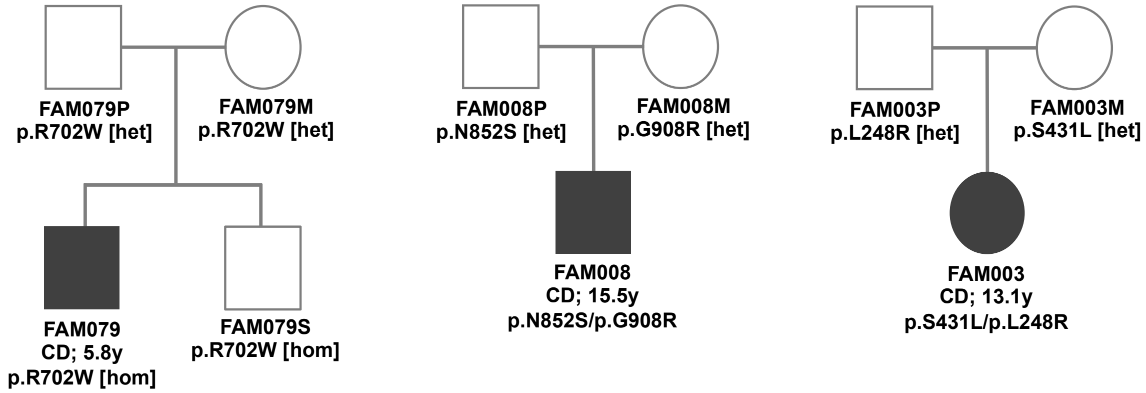

Figure S2. Example pedigrees for homozygous and compound heterozygous *NOD2* carriers in the RGC-GHS DiscovEHR cohort. Diagnosis indicated for the affected probands (filled dark grey symbols); unaffected relatives are depicted as unfilled symbols; individuals not sequenced are depicted as light grey symbols.

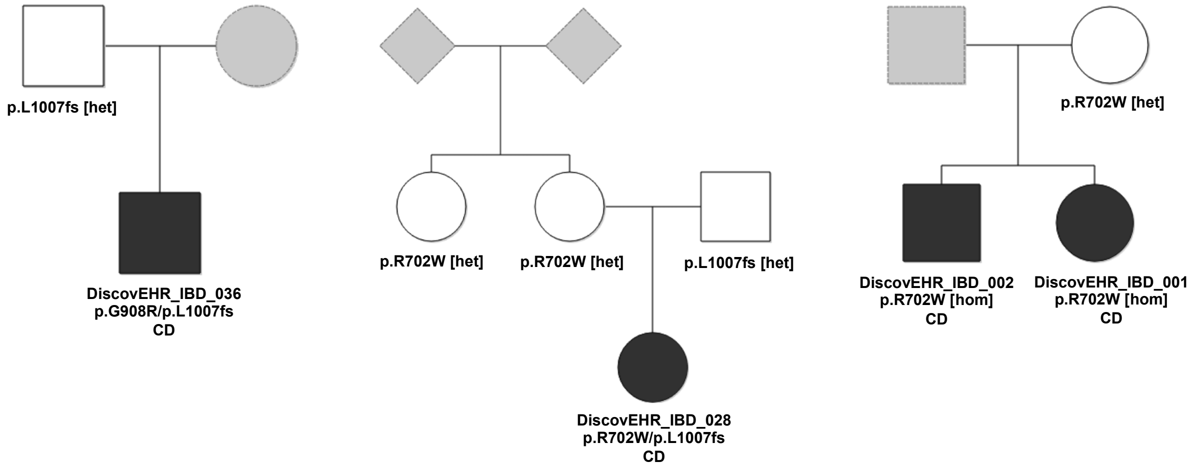

Figure S3. Exome sequencing coverage across the *NOD2* gene. Exon coverage and performance for all exons of the *NOD2* gene using the Nimblegen VCRome 2.1 target design.

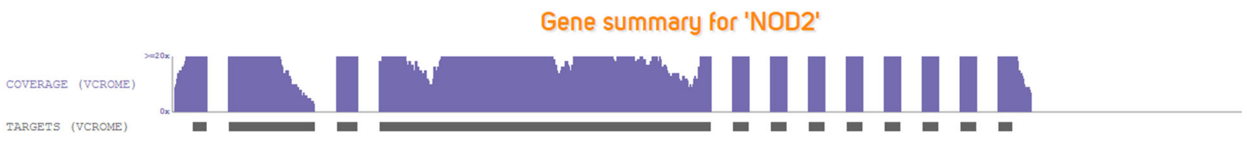

Supplementary Tables:

Table S1. Clinical phenotypes of 92 early onset IBD probands carrying recessive *NOD2* variants.

Table S2. Clinical phenotype summaries for 92 early onset IBD probands carrying recessive *NOD2* variants.

Table S3. Clinical phenotypes of recessive *NOD2* carriers in the RGC-GHS DiscovEHR adult IBD cohort. Cases diagnosed ≤18y are highlighted in dark grey, and diagnosis ≤30y are highlighted in light grey.

Table S4. Genomic coordinates, annotation, frequencies, and bioinformatic prediction scores for *NOD2* variants identified in this study.
